# A Nano-indentation Identification Technique for Viscoelastic Constitutive Characteristics of Periodontal Ligaments

**Published:** 2016-06-01

**Authors:** H. Ashrafi, M. Shariyat

**Affiliations:** 1 Faculty of Mechanical Engineering, University of Kashan, Iran; 2 Faculty of Mechanical Engineering, K. N. Toosi University of Technology, MolaSadra Street, Vanak Square, 19991-43344 Tehran, Iran

**Keywords:** Nano-indentation, Creep and Relaxation, Periodontal Ligaments, Viscoelastic Biological Tissues, Contact Mechanics

## Abstract

**Introduction:**

Nano-indentation has recently been employed as a powerful tool for determining the mechanical properties of biological tissues on nano and micro scales. A majority of soft biological tissues such as ligaments and tendons exhibit viscoelastic or time-dependent behaviors. The constitutive characterization of soft tissues is among very important subjects in clinical medicine and especially, biomechanics fields. Periodontal ligament plays an important role in initiating tooth movement when loads are applied to teeth with orthodontic appliances. It is also the most accessible ligament in human body as it can be directly manipulated without any surgical intervention. From a mechanical point of view, this ligament can be considered as a thin interface made by a solid phase, consisting mainly of collagen fibers, which is immersed into a so-called ground substance. However, the viscoelastic constitutive effects of biological tissues are seldom considered rigorous during Nano-indentation tests.

**Methods:**

In the present paper, a mathematical contact approach is developed to enable determining creep compliance and relaxation modulus of distinct periodontal ligaments, using constant–rate indentation and loading time histories, respectively. An adequate curve-fitting method is presented to determine these characteristics based on the Nano-indentation of rigid Berkovich tips. Generalized Voigt-Kelvin and Wiechert models are used to model constitutive equations of periodontal ligaments, in which the relaxation and creep functions are represented by series of decaying exponential functions of time.

**Results:**

Time-dependent creep compliance and relaxation function have been obtained for tissue specimens of periodontal ligaments.

**Conclusion:**

To improve accuracy, relaxation and creep moduli are measured from two tests separately. Stress relaxation effects appear more rapidly than creep in the periodontal ligaments.

## Introduction

The mechanical characterization of biological tissues is among very important problems in clinical medicine and biomechanics fields. However, complete information regarding the role of mechanical properties of tissues in disease progression, tissues repair, and remodeling mechanisms associated with medical treatments is not available. Tissues are fabricated from complex materials as they are composed of hierarchical structures with unique features on nano or micro scales. Nano-indentation has recently emerged as a powerful tool for determining the mechanical properties of biological tissues on nano and micro scales[1]. This technique has been used to determine the mechanical properties of microstructure, by investigating variations in the mechanical properties with changes in tissue organization or composition in mineralized and soft tissues, and mapping the mechanical properties spatially. In the past, tensile and bending tests have been the most commonly used techniques for measuring mechanical properties of biological tissues[2]. These tests can provide useful information on average bulk mechanical properties, but local or gradient information due to, for example hierarchal structure, cannot be obtained through these tests. Conventional tensile tests cannot be used directly for determining the viscoelastic moduli of objects with small dimensions such as thin films deposited on substrates, new gradient materials whose moduli vary spatially, nano-composites, and heterogeneous biological tissues such as human ligaments and tendons with substantial variations in mechanical properties. Numerous researchers have recently employed Nano-indentation to determine the mechanical properties of biological tissues. Development of post-processing techniques for analyzing indentation data analysis increases advantages of the approach in characterization of biological tissues. However, during Nano-indentation of biological tissues, viscoelastic effects are seldom considered rigorous, in spite of their significant influences.

A periodontal ligament is a thin layer of soft tissue that connects the root of a tooth to the surrounding alveolar bone. Periodontal ligaments play an important role in initiating tooth movement when loads are applied to teeth through orthodontic appliances. It is also the most accessible ligament in a human body as it can be directly manipulated without any surgical intervention. From a mechanical point of view, this ligament can be considered as a thin interface fabricated from a solid phase, consisting mainly of collagen fibers, which is immersed into a so-called ground substance.

Passive tensile ligament tissues are also composed mainly of water and collagen, but contain very little amount of proteoglycans giving cartilage its unique mechanical properties. In keeping with the functional role of these tissues, collagen fibrils are organized primarily in long strands parallel to the axis of loading. Collagen fibrils, which may be hollow tubes, combine in a hierarchical structure, with 20–40 *nm* fibrils being bundled into 0.2–12 *μm *fibers[3]. These fibers are birefringent under polarized light, reflecting an underlying wave or crimp structure with a periodicity between 20 and 100 *μm*. Fibers are bundled into fascicles supported by fibroblasts or tenocytes, and surrounded by a fascicular membrane. Finally, multiple fascicles are bundled into a complete ligament encased in a reticular membrane. Individual collagen fibrils also display some inherent viscoelasticity. This feature is considered to determine the viscoelastic properties of passive tensile tissues.

A majority of body tissues such as ligaments and tendons exhibit viscoelastic or time-dependent behaviors[2-4]. Creep and relaxation are important features of tissue behavior. Investigation of these features requires employing accurate approaches. When these tissues are held at a constant strain level, stresses in tissues decrease. This phenomenon is called stress relaxation. Conversely, when tissues are held at a constant stress level, strains of these tissues increase. This evidence is known as creep[5-7]. An optimal experiment extracts the maximum amount of useful information from specimen being tested. This may often require performing multiple experiments, such as testing at various strain levels or strain rates to robustly capture the true behavior. In relaxation tests, specimen is subjected to a certain strain and the resulting stress is measured as a function of time. In a creep test, specimen is subjected to a constant stress, and the corresponding strain is measured as a function of time.

Lu et al.[8] developed analytical methods to measure creep compliance of some solid polymers and determine creep function in time domain through Nano-indentation method. Odegard et al.[9] investigated dynamic viscoelastic behavior of several polymers at different amplitudes and frequencies of harmonic loading. Huang and Lu[10] developed an approach to directly determine the relaxation function of polymers, using Nano-indentation technique. An analytical method was developed by Huang and Lu[11] to determine two independent viscoelastic functions using a Berkovich axisymmetric nano-indenter. Viscoelastic behavior was observed and studied in particular cartilages and ligaments. Toms et al.[12] studied quasi linear viscoelastic behaviors of human ligaments experimentally. Viscoelastic behavior of articular cartilage was observed and studied by Garcia and Cortes[13] Komatsu et al.[14] studied stress relaxation of ligaments for different deformations. Marangalou et al.[15] developed a modified superposition model to describe viscoelastic behavior of periodontal ligament using conventional tensile tests. Ashrafi and Farid[16] measured the mechanical properties of bones and teeth using a Nano-indentation method.

The scope of this paper is to determine viscoelastic creep and relaxation moduli of biological soft tissues as mechanical attributes, resistance to mechanical injury as a result of both static and dynamic loading, and the force-deformation characteristics of ligaments and tendons. A novel mathematical contact analysis is developed to model viscoelastic creep and relaxation behaviors of periodontal ligaments. In the present paper, it is observed that effects of stress relaxation appear more rapidly than the creep, in periodontal ligaments. Generalized Voigt-Kelvin and Wiechert models are used to model constitutive equations of ligaments wherein the relaxation and creep functions are represented by series of decaying exponential functions of time

### Mathematicla Modeling

A human tooth is secured to alveolar bone by fibrous connective tissues that constitute periodontal ligament. Human periodontal ligament stabilizes teeth in the bone and provides nutritive, proprioceptive, and reparative functions. It is composed of collagenous fibers and a gelatinous ground substance including cells and neurovascular tissues[3]. The fluid pressure has a smaller role in these tissues when loaded in tension and complicating of biphasic model is generally unnecessary. To model segmental mechanics, it is basically sufficient to treat these structures based on one-dimensional approximation[17].

Derivation of the required equations for determining viscoelastic moduli is accomplished under a constant–rate indentation and loading time histories. Generally, Nano-indentation procedure consists of loading, holding, and unloading phases. The diamond Berkovich indenter is modeled as a rigid conical indenter with a half-cone angle of 70.3˚ based on contact cross-sectional area as a function of depth equal to that of conical indenter[1]. During Nano-indentation process, an indenter that may be a spherical or a conical one penetrates specimen’s surface with indentation load *P *and indentation amount *h* that can be recorded as a function of time.

#### Modeling of Relaxation 

**Figure 1:**
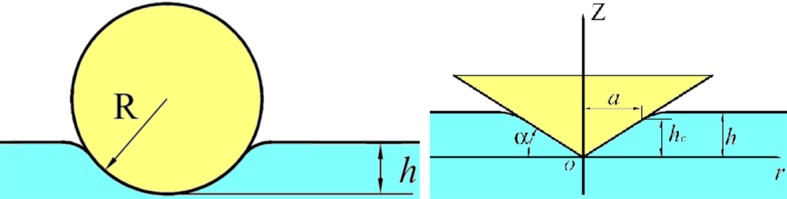
The geometric parameters of the contact of the spherical and conical indenters.

For indentation of a linear elastic, isotropic, and homogeneous half–space, shown in Figure 1, by a rigid conical indenter indenting, Sneddon[18] proposed using the following load–indentation relation:

$$P=\frac{2}{\pi (1\;\;v^{2})\tan\alpha }{Eh}^{2}\;\;(1)$$

where α is the angle between half-space and conical generators and h is the indentation value. In present research, value of this angle is adopted for conical indenter that represents Berkovich configuration, as α=19.7˚. For a contact between a spherical indenter and a linearly elastic, isotropic, and homogeneous half–space, Hertz contact law may be used to relate the load and indentation values:

$$P=\frac{4\sqrt{R}}{3(1-v^{2})}{Eh}^{1.5}\;\;(2)$$

where *R* is the radius of the spherical indenter.

Generally, viscoelastic contact solution can be derived from corresponding elastic contact problem using elastic-viscoelastic correspondence theorem if the displacement-based boundary condition doesn’t vary with time. However, the contact area between indenter and the viscoelastic testing specimen changes with time. Such time-varying boundary value problems cannot be solved by correspondence theorem directly. To solve such contact problems, an effective approach, based on introducing an appropriate hereditary integral operator, was provided by Lee and Radok[19] for situations where indentation contact area doesn’t decrease as time elapses. Ting[20] gave a more general approach to solving viscoelastic contact problems. His approach can be applied to any arbitrary time history of the contact area. Ting’s approach reduces to Lee-Radok approach when contact area does not decrease over time

For a linear viscoelastic, isotropic, and homogeneous tissue with a constant Poisson’s ratio *v* , the following load–indentation relation may be used based on Lee–Radok approach, for the conical indenter:

$$P\left(t\right)=\frac{2}{\pi \left(1-v^{2}\right)\tan\alpha }\int_{0}^{t}E(t-\xi )\frac{{dh}^{2}(\xi )}{d\xi }d\xi \;\;(3)$$

It should be reminded that Equation (3) holds only if the contact area between indenter and half–space shouldn’t decrease with time. This condition can be satisfied through using a constant–rate indentation time history. For an indentation process with a constant–rate indentation time history, one has:

h(t)=V_0_t                                          (4)

in which *V*_0_ is a constant indentation velocity. According to Equation (4), Equation (4) may be rewritten as:

Pt=4V02π1-v2 tan⁡α∫0tEt-ξξdξ  (5)

Equation (5) leads to:

$$\int_{0}^{t}E\left(t-\xi \right)\xi d\xi =\frac{\pi \left(1-v^{2}\right)\tan\alpha }{{4V}_{0}^{2}}P\left(t\right)\;(6)$$

Relaxation modulus can be determined as a function of time based on the recorded load–Nano-indentation data and Equation (6). However, at the beginning of contact (e.g., when the indenting depth is less than 50 *nm*), load-Nano-indentation data aren’t accurate. Moreover, it isn’t advised to apply Equation (6) directly to Nano-indentation data because solving integral equation requires several iterations, starting from initial estimates for low depths or small times. A more adequate approach for correlating between Nano-indentation data and load-indentation relation is the use of an appropriate viscoelastic model for the considered tissue. In the current work, general Wiechert model[7] is used to model viscoelastic tissues, choosing following form for the relaxation modulus:

$$E\left(t\right)=E_{\infty }+\sum_{i=1}^{N}E_{i}e^{{-\lambda }_{i}t}\;\;(7)$$

where *E*_∞_ and *E_i_* are the relaxation coefficients and the *λ_i_* parameters are the reciprocals of the relaxation times.

By substituting Equation (7) into Equation (5), one has:

$$P\left(t\right)=\frac{{4V}_{0}^{2}}{\pi \left(1-v^{2}\right)\tan\alpha }\langle \frac{1}{2}E_{\infty }t^{2}+\sum_{i=1}^{N}\left[\frac{E_{i}}{\lambda_{i}}\left(t-\frac{1}{\lambda_{i}}\right)+\frac{E_{i}}{\lambda_{i}}e^{{-\lambda }_{i}t}\right]\rangle \;\;(8)$$

By substituting Equation (4) into Equation (8), one obtains:

$$P\left(t\right)=\frac{4}{\pi \left(1-v^{2}\right)\tan\alpha }\langle \frac{1}{2}E_{\infty }h^{2}+\sum_{i=1}^{N}\left[\frac{E_{i}}{\lambda_{i}}\left(V_{0}h-\frac{V_{0}^{2}}{\lambda_{i}}\right)+\frac{E_{i}}{\lambda_{i}}e^{-\frac{\lambda_{i}h}{V_{0}}}\right]\rangle \;\;(9)$$

Viscoelastic parameters such as relaxation and reciprocals of relaxation times can be determined after fitting Equation (9) on the average load–indentation curve extracted from Nano-indentation tests. Next, these parameters can be used in Equation (7) to determine relaxation modulus.

Nano-indentation load–indentation relation for a contact between a spherical indenter and a linear viscoelastic, isotropic and homogeneous tissue can be derived by a similar procedure

#### Creep Modelling

For indentation of a homogeneous, linearly elastic, and isotropic half–space by a rigid conical Berkovich indenter, Sneddon[18] derived the relation between contact load and indentation depth as:

$$P=\frac{4}{\pi \left(1-v\right)\tan\alpha }{Gh}^{2\;}(10)$$

where *G* is the shear modulus.

Applying the approach of Lee and Radok[19] to Equation (10) leads to the following time–dependent indentation depth under prescribed indentation loading history, for the linear viscoelastic tissues:

$$h^{2}\left(t\right)=\frac{\pi \left(1-v\right)\tan\alpha }{4}\int_{0}^{t}J\left(t-\xi \right)\left[\frac{dP\left(\xi \right)}{d\xi }\right]d\xi \;(11)$$

where *J*(*t*) is the creep compliance at time t. Equations (11) may be used to find creep compliance *J*(*t*) under a prescribed loading time history, for conical nano-indenters.

For Nano-indentation under a ramp loading at a constant–rate loading, one has:

$$P\left(t\right)=\vartheta_{0}tH\left(t\right)\;(12)$$

in which *ϑ*_0_ and *H*(*t*) are the loading rate and Heaviside unit step function[21], respectively.

By substituting Equation (12) into Equation (11), one finds:

$$h^{2}\left(t\right)=\frac{\pi (1-v)\vartheta_{0\tan\alpha }}{4}\int_{0}^{t}J\left(t-\xi \right)d\xi \;(13)$$

Creep compliance as a function of time can be determined by means of recorded load, Nano-indentation data and Equation (13). However, as mentioned before, at the initial times of contact process (e.g., when indenting depth is less than 50 *nm*), the load–Nano-indentation data aren’t accurate. Furthermore, a proper approach for finding a correlation between indentation data and load-indentation relation is using an appropriate viscoelastic model for considered tissues. A generalized Voigt–Kelvin model[7] is adopted in the present research to model the creep behavior of viscoelastic tissues. Creep compliance of a generalized Voigt–Kelvin model may be written as:

$$J\left(t\right)=J_{0+}\sum_{i=1}^{N}J_{i}(1-e^{-t/\tau_{i}})\;(14)$$

where *J*_0_ , *J_i_* are the compliance coefficients and τi are the retardation times.

By substituting Equation (14) into Equation (13) for the conical indenters, one obtains:

$$h^{2}\left(t\right)=\frac{\pi }{4}v_{0}\left(1-v\right)\tan\alpha \left\{(J_{0}+\sum_{i=1}^{N}J_{i})t-\sum_{i=1}^{N}J_{i}\tau_{i}\left[1-e^{-\frac{t}{\tau_{i}}}\right]\right\}\;(15)$$

By substituting Equation (12) into Equation (15), one has:

$$h^{2}\left(t\right)=\frac{\pi }{4}\left(1-v\right)\tan\alpha \left\{(J_{0}+\sum_{i=1}^{N}J_{i})P(t)-\sum_{i=1}^{N}J_{i}(v_{0}\tau_{i})\left[1-e^{-[\frac{P\left(t\right)}{v_{0}\tau_{i}}]}\right]\right\}\;(16)$$

Compliance values and retardation times are determined after fitting Equation (16) to average load–indentation curve extracted from Nano-indentation tests. Then, relevant viscoelastic parameters of generalized Voigt–Kelvin model are incorporated into Equation (14) to determine the creep compliance of the tissue.

#### The Experimental Set-up And Procedure

**Figure 2:**
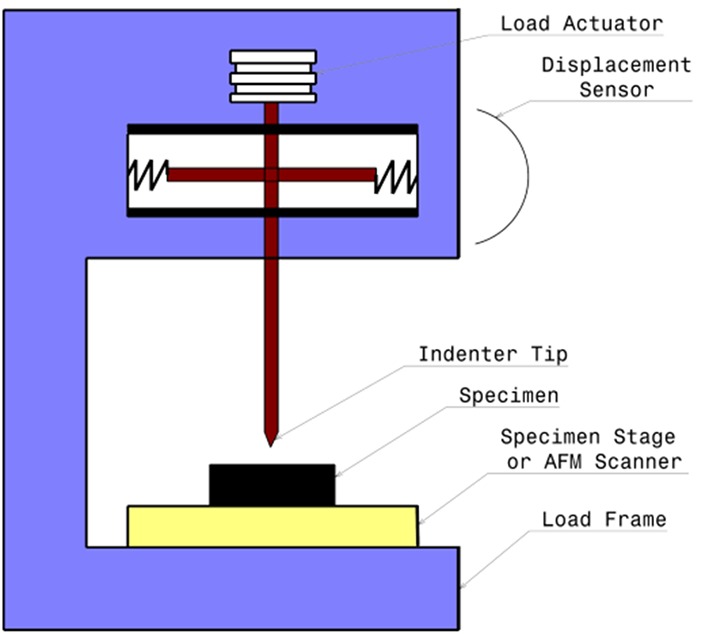
Sketch of the employed nanoindenter machine

The aforementioned procedure is implemented for determining the viscoelastic moduli of biological soft tissues. Tissue specimens of periodontal ligaments were chosen as a case study. The type of required equipment for determination of mechanical properties depends on the required post-processing procedure. Samples used for mechanical measurement were carefully prepared and stored to avoid damage to specimens and degradation of properties. In commercial nano-indenters, displacement is typically controlled by monitoring the capacitance or inductance, while force actuation is provided through electrostatic force generation, magnetic coils, or expansion of a piezoelectric element[1]. A schematic of a nano-indenter machine including a three-plate capacitor for displacement sensing, is shown in Figure 2. A conical tip is mounted directly onto the middle plate of the capacitor and then a load is applied to press the tip into the specimen.

**Figure 3:**
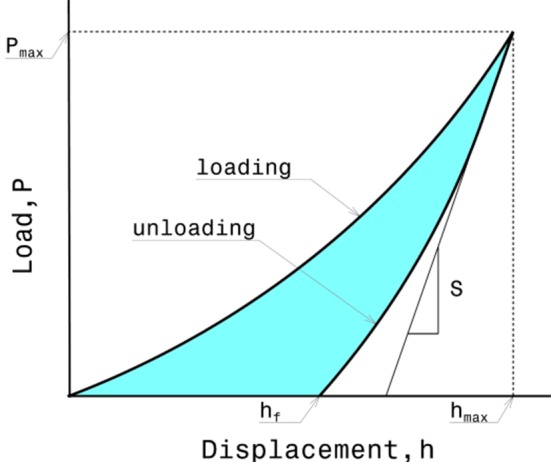
Parameters of the load-displacement (indentation) curve

During Nano-indentation process, the load and displacement are monitored continuously. The result is the load–indentation curve, as demonstrated in Figure 3. The employed Berkovich indenter tip is a conical indenter with half–cone angle of 70.3˚. Each test didn’t start unless the drift rate of indenter tip had dropped below a prescribed value to ensure that a thermal equilibrium condition between specimen and nano-indenter device had been reached. This procedure is necessary as the precision of nano-indenter depends to a great extent on the temperature gradient of instrumentation. When a contact is established between indenter tip and specimen surface, a constant–rate displacement feed and loading is applied. Both load and displacement have been recorded simultaneously at a sampling rate of five data points per second. To allow the displacement or load to follow a linear function of time, continuous stiffness module has to be activated during indentation process. Nano-indentation and load rates were chosen as 5 *nm*/*s*, and Poisson’s ratio was constant and equal to 0.3.

Viscoelastic constitutive behaviors of periodontal ligaments were modeled by a general Wiechert relaxation and by a generalized Voigt–Kelvin creep model. Employing adequate combinations of the two fundamental elements of massless Hookean linear spring and Newtonian dashpot is possible to model various viscoelastic behaviors. A real biological tissue doesn’t relax or retard with a single relaxation or retardation time as predicted by a Maxwell or Kelvin model. Molecular segments with varying lengths contribute to relaxation or retardation, with simpler and shorter segments relaxing or retarding much more quickly than longer ones. This leads to a distribution of relaxation or retardation times, which in turn produces a relaxation or retardation spread over a much longer time than could be modeled accurately with a single relaxation or retardation time. Therefore, to take these issues into account, Wiechert and generalized Voigt–Kelvin models can be constructed using many spring-dashpot Maxwell and Kelvin elements, respectively in order to approximate distribution of relaxation and retardation more precisely. Thus the resulted relaxation and creep moduli may be represented by suitable series of exponential terms. Viscoelastic properties of biological tissues were obtained from stress relaxation and creep tests performed on ten specimens, which were prepared from ten distinct periodontal ligaments. Tissue specimens were first compressed to 10% strain level at a 5 nm/s loading rate using a nano-indenter machine and then held at that strain level for 1000 s. The resulting stress relaxation curves of the 10 specimens were averaged, and then a Wiechert model has been used to fit a curve to the average stress relaxation curve *E*(*t*). In a similar manner, creep tests were performed to obtain uniaxial creep compliance *J*(*t*).

## Results and Conclusions

**Figure 4:**
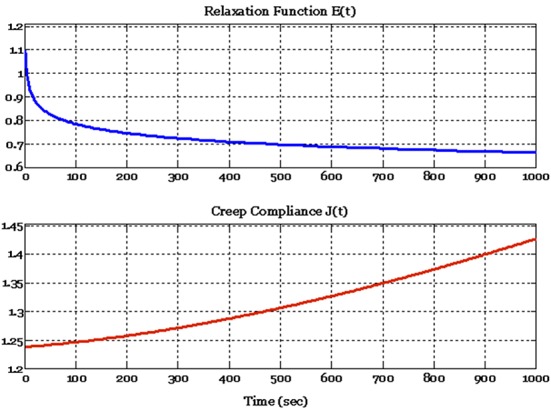
The viscoelastic characteristic relaxation and creep functions of the periodontal ligaments.

Time-dependent creep compliance and relaxation function have been obtained for tissue specimens of periodontal ligaments, using Equation (9) and (16) and experimental Nano-indentation data shown in Figure 4. Viscoelastic parameters such as relaxation or retardation values and retardation or reciprocals of relaxation times were determined after fitting Equations (9) or (16) on average load–indentation curves obtained from Nano-indentation tests. Then, these parameters were used in Equations (7) or (14) to determine relaxation modulus or creep compliance. It is observed that viscoelastic creep compliance is an increasing function of time and viscoelastic relaxation function is a decreasing function of time. Furthermore, stress relaxation effects appear more rapidly than creep in periodontal ligaments.

Mechanical behaviors of biological soft tissues depend upon both rate and duration of the applied stress. Periodontal ligaments exhibit a time-dependent behavior. Viscoelasticity is a result of the losses of internal energy due to internal frictions of microstructure or a fluid flow-induced damping during deformation. The proposed mathematical approach is capable of determination of viscoelastic moduli based on creep results and relaxation based on Nano-indentation tests under a constant-rate loading and displacement (indentation) time histories, respectively. By selecting appropriate tip geometry and testing procedure, a variety of mechanical properties can be measured. This procedure can also be applied to characterize local behaviors of inhomogeneous biological tissues, in which mechanical properties vary spatially. 
